# Cytokine gene regulation by PGE_2_, LTB_4_ and PAF

**DOI:** 10.1155/S0962935192000024

**Published:** 1992

**Authors:** M. Rola-Pleszczynski, J. Stankova

**Affiliations:** 1 Immunology Division Department of Pediatrics Faculty of Medicine University of Sherbrooke Sherbrooke QC J1H 5N4 Canada

## Abstract

The initial response of the host to noxious stimuli produces a nonspecific inflammatory response. A more specific immune response is believed to be modulated by two classes of molecules: lipid mediators (PG, LT and PAF) and cytokines, synthesized by phagocytes and parenchyreal cells. In this review we discuss the increasing evidence of the interrelationship between eicosanoids, PAF and cytokines: IL-1 and TNF induce PG synthesis in various cells and PG, in turn, modulate cytokine production. We focused on the regulatory effects of **LTB**_4_, **PGE**_2_ and PAF on cytokine gene expression.


Mediators of Inflammation 1, 5–8 (1992)

### Cytokine gene regulation by $PGE_2$, $LTB_4$ and PAF

M. Rola-Pleszczynski[CA] and J. Stankova

Immunology Division, Department of Pediatrics, Faculty of Medicine, University of Sherbrooke, Sherbrooke, QC, Canada, J1H 5N4.

[CA] Corresponding Author

**The initial response of the host to noxious stimuli produces a nonspecific inflammatory response. A more specific immune response is believed to be modulated by two classes of molecules: lipid mediators (PG, LT and PAF) and cytokines, synthesized by phagocytes and parenchymal cells. In this review we discuss the increasing evidence of the interrelationship between eicosanoids, PAF and cytokines: IL-1 and TNF induce PG synthesis in various cells and PG, in turn, modulate cytokine production. We focused on the regulatory effects of $LTB_4$, $PGE_2$ and PAF on cytokine gene expression.**

**Key words:** Alveolar macrophages, Cyclic nucleotides, Endothelial cells, IL-1, IL-6, Monocytes, mRNA, Neutrophils, TNF, Transcription

When confronted with a variety of noxious stimuli, the host responds by producing an array of soluble factors and by mobilizing various cell populations. In most instances, the initial response is relatively nonspecific and consists of various degrees of inflammation. Among the soluble mediators which participate in this inflammatory response and which may also modulate the subsequent more specific immune response, two classes of molecules have emerged as the principal protagonists: lipid mediators, derived from cell membrane phospholipids, and cytokines, synthesized by phagocytes and parenchymal cells.

Within minutes of stimulation, eicosanoids such as prostaglandins (PG) and leukotrienes (LT), as well as platelet activating factor (PAF) are produced by the action of phospholipase $A_2$ ($PLA_2$) on the membrane phospholipid 1-0-alkyl-2-arachidonoyl-sn-glycero-3-phosphocholine and subsequent oxidative metabolism of the freed arachidonic acid or acetylation of the remaining 1-alkyl-2-lyso-glycerophosphocholine (lyso-PAF) molecule, respectively. These lipid mediators in turn can act on a variety of cell populations, often, including the cells which produced them.[1] Their bioactions are thought to be mediated by specific cell membrane receptors. Recently, a PAF receptor from guinea-pig lung has been cloned[2] and its proposed structure suggests that it is associated with a G protein. Functional studies had previously suggested that this may also be the case with $LTB_4$ receptors.[3] Postreceptor events and signal transduction pathways in cellular responses triggered by LT, PG and PAF are objects of intense research using metabolic inhibitors and receptor antagonists. In the present review, we will focus on the regulatory effects of $LTB_4$, $PGE_2$ and PAF on cytokine gene expression (Table 1).

**Table 1.** Summary of actions of eicosanoids and PAF on cytokine gene regulations.

| Cytokine | Mediator | Transcription | mRNA | Protein |
|---|---|---|---|---|
| TNFα | $LTB_4$ | | ↑ | ↑ |
| | $PGE_2$ (low dose) | | ↑ | ↑ |
| | $PGE_2$ (high dose) | | ↓ | ↓ |
| | PAF | | ↑ | ↑ |
| IL-1 | $LTB_4$ | ↑ | ↑ | ± |
| | $PGE_2$ | | – | – |
| | PAF | | ↑ | ↑ |
| IL-6 | $LTB_4$ | ↑ | ↑ | ↑ |

### Tumour Necrosis Factor (TNFα)

TNFα is a cytokine produced preferentially by activated macrophages, but also by NK cells and neutrophils. TNFα may play a role in immune modulation[4] and anti-tumour defences,[5] in addition to being a potent mediator for numerous inflammatory responses, such as endotoxic shock, adult respiratory distress syndrome and bowel necrosis.[6–8] In these conditions, however, synergy with other mediators, including PAF, may be needed for expression of disease.

When human monocytes are exposed to graded concentration of $LTB_4$, their cell-free supernatants contain increased amounts of TNFα which peaks at 8–16 h.[9] The maximal effect of $LTB_4$ is observed at concentrations of $10^{-10}$ M. Endogenous lipoxygenase metabolites may also be involved in enhanced TNFα production following some stimuli, such as silica, asbestos,[10] PAF[11] or lipopolysaccharide (LPS).[12] Evidence for such an involvement is derived from use of 5-lipoxygenase (5-LO) inhibitors which can partially or totally block TNFα production. Addition of exogenous $LTB_4$ can restore TNFα production under certain

© 1992 Rapid Communications of Oxford Ltd circumstances.[11] In contrast, inhibition of 5-LO activation using MK-886, which binds the 5-lipoxygenase-activating protein (FLAP), does not affect TNFα production[13] in response to phorbol ester, Concanavalin A, LPS or zymosan. TNFα gene expression can be stimulated by phorbol esters, LPS or TNFα itself. Under these conditions, inhibition of $PLA_2$ by bromophenacyl bromide or quinacrine, or of lipoxygenases by ketoconazole or nordihydroguaiaretic acid (NDGA) results in inhibition of TNFα mRNA accumulation and TNFα gene transcription.[14-16] On the other hand, exogenous $LTB_4$ increases TNFα mRNA.[14] Interestingly, the dual cyclooxygenose (CO)/5-LO inhibitor, tebufelone, at 20–25 μM, inhibits TNFα mRNA accumulation while enhancing TNFα production.[17]

In comparison to 5-LO metabolites, CO metabolites such as $PGE_2$ have been shown to inhibit TNFα production at high concentrations,[18,19] presumably by augmenting cAMP levels in the cells. Low concentrations of $PGE_2$, however, appear to stimulate guanylate cyclase and result in augmented TNFα production.[20,21] TNFα mRNA accumulation is also inhibited by $PGE_2$,[22] an effect associated with decreased TNFα transcription[14] (Fig. 1).

PAF can enhance TNFα production by rat alveolar macrophages[11] and human monocytes[23-25] and myeloid cells.[26] In the macrophages, the activation pathway may involve 5-LO, since inhibitors such as NDGA or AA-861 can abrogate the effect of PAF.[11] In human monocytes and myeloid cell lines, the involvement of the 5-LO pathway in PAF-mediated TNFα production is still unclear. PAF induces, however, a bimodal dose-response pattern in these cells, with both a nanomolar and a femtomolar concentration peak of activation.[26] Enhanced accumulation of TNFα mRNA is maximally stimulated by $10^{-13}$ and $10^{-7}$ M PAF in monocytes and in HL-60 promyelocytic leukemia cells induced by 1,25 $(OH)_2$ vitamin $D_3$ to differentiate into macrophages.[26]

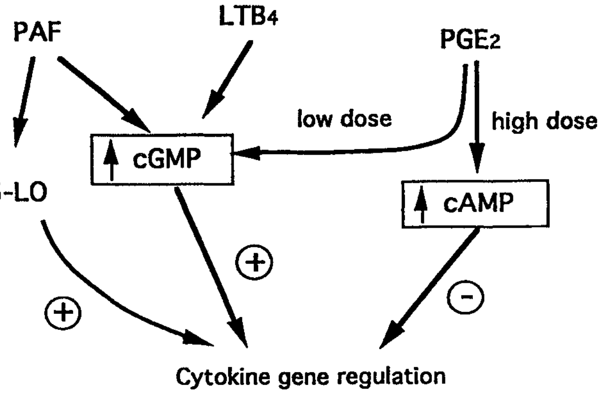

FIG. 1. Schematic representation of cytokine gene regulation by eicosanoids and PAF, involving cyclic nucleotides.

### Interleukin-1 (IL-1)

IL-1 is a key protagonist of immune and inflammatory events, being involved, among other effects, in T-cell activation by accessory cells and in induction of fever and other features of inflammation.[27] It is produced principally by monocytes/macrophages,[28] but also by a variety of other cell types such as B-cells, endothelial cells, keratinocytes, polymorphonuclear (PMN) leukocytes, etc. There are two species of IL-1, designated IL-1α and IL-1β, which share cell receptors and have similar bioactions in spite of being derived from two separate genes.

Addition of exogenous $LTB_4$ to monocytes stimulates IL-1β transcription and mRNA accumulation (Rola-Pleszczynski and Stankova, *in preparation*), but little IL-1 protein release.[29] Earlier reports that IL-1 activity was enhanced in supernatants from $LTB_4$-treated monocytes[30] were consistent with measures of both IL-1 and IL-6 in bioassays of lymphocyte activating factor (LAF) activity in monocyte supernatants.[29,31]

Prostaglandins, on the other hand, have been known for some time to inhibit lymphokine secretion.[32] $PGE_2$ inhibits IL-1 production by monocytes via a post-transcriptional mechanism involving increased cAMP levels within the cell.[33] $PGE_2$ also inhibits secretion of IL-1 by large granular lymphocyte (LGL)[34] but has no direct effect on IL-1 secretion by phagocytic cells of the thymic reticulum[35] or by peritoneal macrophages.[36] While $PGE_2$ inhibits TNFα mRNA accumulation in monocytes, it has no effect on IL-1α or IL-1β mRNA levels.[22]

PAF stimulates IL-1α and IL-1β production in human monocytes in a concentration-dependent manner, in synergy with other stimuli such as LPS, MDP or IFNγ.[24,37-39] As with TNFα, IL-1 production in PAF-treated monocytes follows a bimodal pattern, with peak activities at $10^{-13}$ and $10^{-8}$ M PAF.[24,39] In rats treated with a continuous infusion of PAF via an osmotic mini-pump, IL-1 production by splenic macrophages was enhanced by lower doses of PAF, while higher doses had an inhibitory effect.[40] At the present time, it is unclear whether PAF regulates IL-1α and IL-1β production via transcriptional or post-transcriptional mechanisms. IL-1β mRNA expression in THP-1 cells, however, can be enhanced by $10^{-10}$ M PAF.[41]

### Interleukin-6

IL-6 is a multifunctional cytokine produced by monocytes, macrophages, endothelial cells, fibroblasts, keratinocytes, T-cells and some tumour cells. Its numerous synonyms reflect its various biological activities, as B-cell stimulatory factor 2, interferon

$\beta_2$, hybridoma-plasmacytoma growth factor, hepatocyte-stimulating factor.[42–46] IL-6 is an important regulator of T- and B-cell functions, haematopoiesis and acute phase responses.[47,48] Infectious agents, endotoxin and the inflammatory cytokines TNF$\alpha$ and IL-1 can induce IL-6 production, while dysregulation of IL-6 expression is associated with certain chronic inflammatory, autoimmune and haematopoietic disorders. The findings that IL-6 production is associated with inflammatory states suggests that its production may also be modulated by inflammatory lipid mediators.

When human monocytes are cultured in the presence of graded concentrations of $LTB_4$, a significant stimulation of production of bioactive and immunoreactive IL-6 is observed.[29,31] Nanomolar concentrations of $LTB_4$ are optimal, while the $\omega$-oxidation products 20-OH-$LTB_4$ and 20-COOH-$LTB_4$ are only 22% and 2% effective, respectively. $LTB_4$ induces an accumulation of IL-6 mRNA in treated monocytes with a superposable dose-response, and maximal accumulation at 1 h. While IL-6 mRNA half-life in untreated cells is approximately 1 h, it is extended to 3 h in $LTB_4$-treated monocytes. Moreover, nuclear transcription of IL-6 mRNA is augmented at 30 min by a factor of five in $LTB_4$-treated cells. Furthermore, $LTB_4$-treated monocytes contain a nuclear protein factor (NF) which binds to a promoter region of the IL-6 gene, called NF-IL-6-binding domain, and which may be involved, among other factors, in $LTB_4$-mediated induction of IL-6 gene transcription.[31]

PAF can also stimulate IL-6 production by human monocytes in the picomolar range and this stimulation is enhanced by prior treatment with TNF$\alpha$ or IFN$\gamma$.[49] In contrast, rat alveolar macrophages require a second stimulus, such as LPS or MDP, to respond to PAF with augmented IL-6 production.[50] This augmented production is brought down to baseline by pretreatment of the cells with the FLAP inhibitor MK-886 or the 5-LO inhibitor AA-861, suggesting that under these conditions, the action of PAF is mediated by endogenous 5-LO metabolites. Human PMN and endothelial cells can also respond to PAF with enhanced IL-6 production and IL-6 mRNA accumulation (unpublished).

### Conclusions

There is increasing evidence to suggest that the production of eicosanoids, PAF and cytokines may be interrelated: IL-1 and TNF induce PG synthesis in various cells[51–53] and PG, in turn, modulate cytokine production, as discussed in this review. In contrast, LT can augment IL-1, IL-6 and TNF production and endogenous LT production may

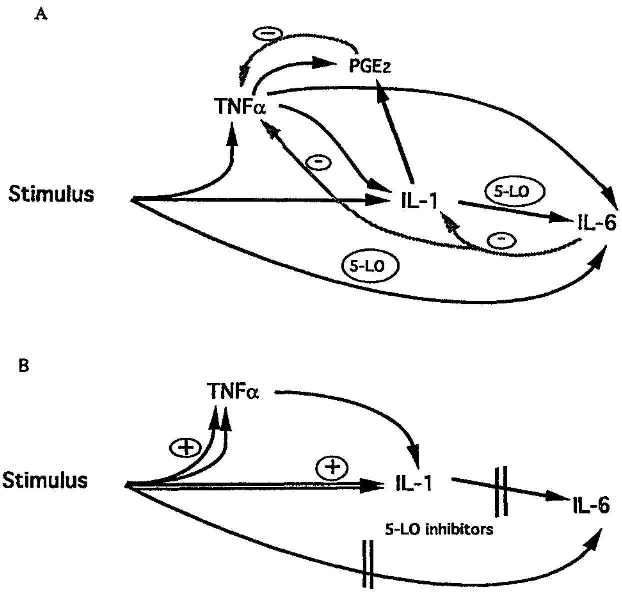

FIG. 2. Schematic representation of mutual regulatory pathways between TNF, IL-1 and IL-6. (A) Proposed preferential involvement of 5-LO in IL-6 production. (B) Proposed mechanism of action of 5-LO inhibitors in suppressing the negative feed-back of IL-6 on TNF and IL-1 production.

play a role in TNF and IL-6 synthesis. IL-1, TNF and IFN can also induce the synthesis of PAF in several cell types, including endothelial cells, neutrophils and macrophages,[54–56] while PAF can, in turn, augment IL-1, IL-6 and TNF production by rat and human cells. Such a positive feedback loop at this level, with potential for amplification of immune or inflammatory responses, may be counterbalanced by the negative feedback action of IL-6 on both IL-1 and TNF.[57,58] This negative feedback may account for the limited production of IL-1 by $LTB_4$-stimulated monocytes which readily produce large amounts of IL-6. It may also explain the augmented production of IL-1 and TNF observed after treatment of monocytes with a dual CO/5-LO inhibitor, a treatment which may preferentially inhibit IL-6 production (Fig. 2).

### References

1. Rola-Pleszczynski M. Immunoregulation by leukotrienes and other lipoxygenase metabolites. *Immunol Today* 1985; **10:** 302–307.
2. Honda Z-I, Nakamura M, Miki I, *et al.* Cloning by functional expression of platelet-activating factor receptor from guinea-pig lung. *Nature* 1991; **349:** 342–346.
3. Molski TFP, Naccache PH, Marsh ML, *et al.* Pertussis toxin inhibits the rise in the intracellular concentration of free calcium that is induced by chemotactic factors in rabbit neutrophils: possible role of the 'G proteins' in calcium mobilization. *Biochem Biophys Res Comm* 1984; **124:** 644–650.
4. Philip R, Epstein LB. Tumor necrosis factor as immunomodulator and mediator of monocyte cytotoxicity induced by itself, gamma-interferon and interleukin 1. *Nature* 1986; **323:** 86–89.
5. Carswell EA, Old LJ, Kassel RL, *et al.* An endotoxin induced serum factor that causes the necrosis of tumors. *Proc Natl Acad Sci USA* 1975; **72:** 3666–3670.
6. Chang SW, Fedderson CO, Henson PM. Platelet-activating factor mediates hemodynamic changes and lung injury in endotoxin-treated rats. *J Clin Invest* 1987; **79:** 1498–1509.
7. Marks JD, Marks CB, Luce JM, *et al.* Plasma tumor necrosis factor in patients with septic shock: mortality rate, incidence of adult respiratory distress syndrome, and effects of methylprednisolone administration. *Am Rev Resp Dis* 1990; **141:** 94–97.

8. Sun X, Hsueh W. Bowel necrosis induced by tumor necrosis factor in rats is mediated by platelet activating factor. *J Clin Invest* 1988; **81:** 1328–1331.
9. Gagnon L, Filion LG, Dubois C, *et al.* Leukotrienes and macrophage activation: augmented cytotoxic activity and enhanced interleukin 1, tumor necrosis factor and hydrogen peroxyde production. *Agents & Actions* 1989; **26:** 141–147.
10. Dubois C, Bissonnette E, Rola-Pleszczynski M. Asbestos fibers and silica particles stimulate rat alveolar macrophages to release tumor necrosis factor: autoregulatory role of leukotriene $B_4$. *Am Rev Resp Dis* 1989; **139:** 1257–1264.
11. Dubois C, Bissonnette E, Rola-Pleszczynski M. Platelet-activating factor (PAF) stimulates tumor necrosis factor production by alveolar macrophages: prevention by PAF receptor antagonists and lipoxygenase inhibitors. *J Immunol* 1989; **143:** 964–971.
12. Schade UF, Ernst M, Reinke M, *et al.* Lipoxygenase inhibitors suppress formation of tumor necrosis factor *in vitro* and *in vivo. Biochem Biophys Res Comm* 1989; **159:** 748–754.
13. Hoffman T, Lee YL, Lizzio EF, *et al.* Absence of modulation of monokine production via endogenous cyclooxygenase or 5-lipoxygenase metabolites: MK-886 (3-[1-(4-chlorobenzyl)-3-t-butyl-thio-5-isopropylondol-2-yl]-2,2-dimethyl-propanoic acid), indomethacin, or arachidonate fail to alter immunoreactive interleukin 1$\beta$, or TNF-$\alpha$ production by human monocytes *in vitro. Clin Immunol Immunopathol* 1991; **58:** 399–408.
14. Horiguchi J, Spriggs D, Imamura K, *et al.* Role of arachidonic acid metabolism in transcriptional induction of tumor necrosis factor gene expression by phorbol ester. *Mol Cell Biol* 1989; **9:** 252–258.
15. Mohri M, Spriggs DR, Kufe D. Effects of lipopolysaccharide on phospholipase $A_2$ activity and tumor necrosis factor expression in HL-60 cells. *J Immunol* 1990; **144:** 2678–2682.
16. Spriggs DR, Sherman ML, Imamura K, *et al.* Phospholipase $A_2$ activation and autoinduction of tumor necrosis factor gene expression by tumor necrosis factor. *Cancer Res* 1990; **50:** 7101–7107.
17. Sirko SP, Schindler R, Doyle MJ, *et al.* Transcription, translation and secretion of interleukin 1 and tumor necrosis factor: effects of tebufelone, a dual cyclooxygenase/5-lipoxygenase inhibitor. *Eur J Immunol* 1991; **21:** 243–250.
18. Renz H, Gong J-H, Schmidt A, *et al.* Release of tumor necrosis factor-$\alpha$ from macrophages. Enhancement and suppression are dose-dependently regulated by prostaglandin $E_2$ and cyclic nucleotides. *J Immunol* 1988; **141:** 2388–2393.
19. Kunkel SL, Spengler M, May MA, *et al.* Prostaglandin $E_2$ regulates macrophage-derived tumor necrosis factor gene expression. *J Biol Chem* 1988; **263:** 5380–5384.
20. Gong J-H, Renz H, Sprenger H, *et al.* Enhancement of tumor necrosis factor-$\alpha$ gene expression by low doses of prostaglandin $E_2$ and cyclic GMP. *Immunobiol* 1990; **182:** 44–55.
21. Kovacs EJ, Radzioch D, Young HA, *et al.* Differential inhibition of IL-1 and TNF$\alpha$ mRNA expression by agents which block second messenger pathways in murine macrophages. *J Immunol* 1988; **141:** 3101–3105.
22. Scales WE, Chensue SW, Otterness I, *et al.* Regulation of monokine gene expression: prostaglandin $E_2$ suppresses tumor necrosis factor but not interleukin-1$\alpha$ or $\beta$-mRNA and cell-associated bioactivity. *J Leuk Biol* 1989; **45:** 416–421.
23. Rola-Pleszczynski M, Bossé J, Bissonnette E, *et al.* PAF-acether enhances the production of tumor necrosis factor by human and rodent lymphocytes and macrophages. *Prostaglandins* 1988; **35:** 802.
24. Poubelle P, Gingras D, Demers C, *et al.* Platelet activating factor (PAF-acether) enhances the concomitant production of tumor necrosis factor alpha and interleukin-1 by subsets of human monocytes. *Immunol* 1991; **72:** 181–187.
25. Bonavida B, Braquet P. Effect of platelet-activating factor (PAF) on monocyte activation and production of tumor necrosis factor (TNF). *Prostaglandins* 1988; **35:** 802.
26. Rola-Pleszczynski M, Stankova J. Differentiation-dependent modulation of TNF production by PAF in human HL-60 myeloid leukemia cells. *J Leuk Biol* 1992; in press.
27. Dinarello C. Biology of interleukin-1. *FASEB J* 1988; **2:** 108–115.
28. Le J, Vilcek J. Tumor necrosis factor and interleukin-1: cytokines with multiple overlapping biological activities. *Lab Invest* 1987; **56:** 234–248.
29. Poubelle P, Stankova J, Grassi J, *et al.* Leukotriene $B_4$ up-regulates IL-6 rather than IL-1 synthesis in human monocytes. *Agents & Actions* 1991; **34:** 42–45.
30. Rola-Pleszczynski M, Lemaire I. Leukotrienes augment interleukin-1 production by human monocytes. *J Immunol* 1985; **135:** 3958–3961.
31. Stankova J, Rola-Pleszczynski M. Interleukin-6 production by mononuclear phagocytes can be stimulated by leukotrienes. *Arch Immunol Therap Exp* 1991; in press.
32. Gordon D, Bray MA, Morley J. Control of lymphokine secretion by prostaglandins. *Nature* 1976; **262:** 401–403.
33. Knudsen PJ, Dinarello C, Strom TB. Prostaglandins post-transcriptionally inhibit monocyte expression of interleukin-1 activity by increasing cyclic adenosine monophosphate. *J Immunol* 1986; **137:** 3187–3194.
34. Herman J, Rabson AR. Prostaglandin $E_2$ depresses natural cytotoxicity by inhibiting interleukin-1 production by large granular lymphocytes. *Clin Exp Immunol* 1984; **57:** 380–384.
35. Papiernik M, Homo-Delarche F. Thymic reticulum in mice. III Phagocytic cells of the thymic reticulum in culture secrete both prostaglandin $E_2$ and interleukin-1 which regulate thymocyte proliferation. *Eur J Immunol* 1983; **13:** 689–692.
36. Hayari Y, Kukulansky T, Globerson A. Regulation of thymocyte proliferation response by macrophage-derived prostaglandin $E_2$ and interleukin-1. *Eur J Immunol* 1985; **15:** 43–47.
37. Ward SG, Lewis GP, Westwick J. Platelet-activating factor stimulates interleukin-1 production by human adherent monocytes-macrophages. In: P Braquet, Ed. *New Trends in Lipid Mediator Research.* Basel:Karger, 1988: 41.
38. Salem P, Derickx S, Dulioust A, *et al.* Immunoregulatory functions of paf-acether. Enhancement of IL-1 production by muramyl dipeptide-stimulated monocytes. *J Immunol* 1990; **144:** 1338–1344.
39. Barthelson R, Valone F. Interaction of platelet-activating factor with interferon-$\gamma$ in the stimulation of interleukin-1 production by human monocytes. *J Allergy Clin Immunol* 1990; **86:** 193–201.
40. Pignol B, Hénane S, Sorlin B, *et al.* Effect of long-term treatment with platelet-activating factor on IL-1 and IL-2 production by rat spleen cells. *J Immunol* 1990; **145:** 980–984.
41. Barthelson RA, Valone FH. Platelet-activating factor stimulates expression of IL-1 beta mRNA in THP-1 cells. *Lipids* 1991: **26:** 257–260.
42. Hirano T, Yasukawa K, Harada H, *et al.* Complementary DNA for a novel human interleukin (BSF-2) that induces B-lymphocytes to produce immunoglobulin. *Nature* 1986; **324:** 73-78.
43. Poupart P, Vandenabeele P, Cayphas S, *et al.* B-cell growth modulating and differentiating activity of recombinant human 26-kd protein (BSF-2, IFN-$\beta_2$, HPGF). *EMBO J* 1987; **6:** 1219–1224.
44. Gauldie J, Richards C, Harnish D *et al.* Interferon $\beta_2$/B-cell stimulatory factor type 2 shares identity with monocyte-derived hepatocyte-stimulating factor and regulates the major acute phase protein response in liver cells. *Proc Natl Acad Sci USA* 1987; **84:** 7251–7255.
45. Sehgal PB, Helfgott DC, Santhanam U, *et al.* Regulation of the acute phase and immune responses in viral disease: enhanced expression of the '$\beta_2$-interferon/hepatocyte-stimulating factor-interleukin-6' gene in virus-infected human fibroblasts. *J Exp Med* 1988; **167:** 1951–1962.
46. Utsumi K, Takai Y, Tada T *et al.* Enhanced production of IL-6 in tumor-bearing mice and determination of cells responsible for its augmented production. *J Immunol* 1990; **145:** 397–403.
47. Garman RD, Jacobs KA, Clark SC *et al.* B-cell-stimulatory factor 2 ($\beta_2$ interferon) functions as a second signal for interleukin-2 production by mature murine T-cells. *Proc Natl Acad Sci USA* 1987; **84:** 7629–7633.
48. Wong GG, Clark SC. Multiple actions of interleukin-6 within a cytokine network. *Immunol Today* 1988; **9:** 137–139.
49. Rola-Pleszczynski M, Bouvrette L, Thivierge M, *et al.* Platelet-activating factor enhances interleukin-6 production by monocytes, alveolar macrophages and endothelial cells. *FASEB J* 1990; **4:** A1713.
50. Thivierge M, Rola-Pleszczynski M. Modulation of IL-6 production by pulmonary macrophages in response to PAF-acether. *Cytokine* 1991; submitted.
51. Mizel SB, Dayer J-M, Krane SM, *et al.* Stimulation of rheumatoid synovial cell collagenase and prostaglandin production by partially purified lymphocyte-activating factor (interleukin-1). *Proc Natl Acad Sci USA* 1981; **78:** 2474–2477.
52. Albrightson CR, Baenziger NL, Needleman P. Exaggerated human vascular cell prostaglandin biosynthesis mediated by monocytes; role of monokines and interleukin-1. *J Immunol* 1985; **135:** 1872–1877.
53. Bachwich PR, Chensue SW, Larrick JW, *et al.* Tumor necrosis factor stimulates interleukin-1 and prostaglandin $E_2$ production in resting macrophages. *Biochem Biophys Res Comm* 1986; **136:** 94–101.
54. Camussi G, Bussolino F, Salvidio G, *et al.* Tumor necrosis factor/cachectin stimulates peritoneal macrophages, polymorphonuclear neutrophils and vascular endothelial cells to synthesize and release platelet-activating factor. *J Exp Med* 1987; **166:** 1390–1404.
55. Bussolino F, Tetta C, Breviario F, *et al.* Interleukin-1 stimulates platelet-activating factor production in cultured human endothelial cells. *J Clin Invest* 1986; **77:** 2027–2033.
56. Valone FH, Epstein LB. Biphasic platelet activating factor synthesis by human monocytes stimulated with IL-1 beta, tumor necrosis factor or IFN-gamma. *J Immunol* 1988; **141:** 3945–3950.
57. Schindler R, Mancilla J, Endres S *et al.* Correlations and interactions in the production of interleukin-6 (IL-6), IL-1, and tumor necrosis factor (TNF) in human blood mononuclear cells: IL-6 suppresses IL-1 and TNF. *Blood* 1990; **75:** 40–47.
58. Aderka D, Le J, Vilcek J. IL-6 inhibits lipopolysaccharide-induced tumor necrosis factor production in cultured human monocytes, U937 cells, and in mice. *J Immunol* 1989; **143:** 3517–3523.

ACKNOWLEDGEMENTS. Studies by the authors were supported by grants from the Medical Research Council of Canada, the National Cancer Institute and by Scholarships from the Fonds de Recherche en Santé du Québec.

**Received 16 October 1991; accepted 29 October 1991**
